# The influence of the biological pump on ocean chemistry: implications for long‐term trends in marine redox chemistry, the global carbon cycle, and marine animal ecosystems

**DOI:** 10.1111/gbi.12176

**Published:** 2016-02-29

**Authors:** K. M. Meyer, A. Ridgwell, J. L. Payne

**Affiliations:** ^1^ Department of Earth and Environmental SciencesWillamette UniversitySalemORUSA; ^2^ Department of Earth SciencesUniversity of CaliforniaRiversideCAUSA; ^3^ School of Geographical SciencesUniversity of BristolBristolUK; ^4^ Department of Geological SciencesStanford UniversityStanfordCAUSA

## Abstract

The net export of organic matter from the surface ocean and its respiration at depth create vertical gradients in nutrient and oxygen availability that play a primary role in structuring marine ecosystems. Changes in the properties of this ‘biological pump’ have been hypothesized to account for important shifts in marine ecosystem structure, including the Cambrian explosion. However, the influence of variation in the behavior of the biological pump on ocean biogeochemistry remains poorly quantified, preventing any detailed exploration of how changes in the biological pump over geological time may have shaped long‐term shifts in ocean chemistry, biogeochemical cycling, and ecosystem structure. Here, we use a 3‐dimensional Earth system model of intermediate complexity to quantitatively explore the effects of the biological pump on marine chemistry. We find that when respiration of sinking organic matter is efficient, due to slower sinking or higher respiration rates, anoxia tends to be more prevalent and to occur in shallower waters. Consequently, the Phanerozoic trend toward less bottom‐water anoxia in continental shelf settings can potentially be explained by a change in the spatial dynamics of nutrient cycling rather than by any change in the ocean phosphate inventory. The model results further suggest that the Phanerozoic decline in the prevalence ocean anoxia is, in part, a consequence of the evolution of larger phytoplankton, many of which produce mineralized tests. We hypothesize that the Phanerozoic trend toward greater animal abundance and metabolic demand was driven more by increased oxygen concentrations in shelf environments than by greater food (nutrient) availability. In fact, a lower‐than‐modern ocean phosphate inventory in our closed system model is unable to account for the Paleozoic prevalence of bottom‐water anoxia. Overall, these model simulations suggest that the changing spatial distribution of photosynthesis and respiration in the oceans has exerted a first‐order control on Earth system evolution across Phanerozoic time.

## Introduction

A complex set of physical and biological processes mediate the transfer of fixed carbon from the marine photic zone to deeper waters and the seafloor. As a result of this ‘biological pump’, nutrient concentrations are depleted in surface waters where there is net photosynthesis and elevated at depth where there is net respiration (Sigman & Haug, 2003; Sarmiento & Gruber, 2006). Primary production rates are highest in upwelling zones, where nutrient‐rich deep waters are transported to the surface (Ryther, 1969; Martin *et al*., 1987). Respiration at depth leads to oxygen depletion in oxygen minimum zones, with [O_2_] far from equilibrium with the atmosphere. The interplay between the biological pump and ocean circulation thus generates important spatial gradients in nutrients, oxygen, and dissolved inorganic carbon in the modern ocean (Sarmiento & Gruber, 2006).

The biological pump has likely changed over the course of the Phanerozoic, and its evolution may have played an important role in the coevolution of biogeochemical cycles and the marine biosphere. In this study, we attempt to place quantitative constraints on the role of these biological pump changes in controlling the evolution of marine ecosystems by focusing on two key components of the biological pump, biological pump strength and remineralization depth. The strength of the biological pump refers to the flux of fixed carbon exiting the surface ocean, which provides an upper limit on the amount of organic matter than can be transported to depth and influences the oxygen demand within the ocean's interior. Remineralization depth is the distance below the sea surface at which the sinking organic carbon is oxidized. Both components of the biological pump impact the distribution of oxygen and nutrients within the deep ocean. Numerical models often characterize the remineralization depth as an e‐folding length scale, that is, the depth by which the proportion 1/e, or ~37%, of the organic carbon exported from the surface ocean remains (Kwon *et al*., 2009). In the oceans, the surface ocean primary production is determined by the concentration of the limiting nutrient and light availability. The remineralization depth is determined by the factors influencing the sinking rate of organic matter, such as mineral ballast, water viscosity, cell size, particle aggregation, food web processes, and the relative lability or recalcitrance of the organic molecules.

The biological pump also exerts a strong control on the taxonomic composition and ecological structure of the marine biosphere through its effects on the spatial distributions of nutrients, fixed carbon, and dissolved oxygen (e.g., Levin, 2003; Rex *et al*., 2006; Sperling *et al*., 2014). For example, phytoplankton blooms tend to be focused in areas of upwelling due to the high nutrient content of deep waters (e.g., de la Rocha, 2006). Also, large benthic animals are much more common in shallow water settings where both food and oxygen are available at high concentrations (e.g., Rex *et al*., 2006). Oxygen limitation tends to exclude them from oxygen minimum zones (Levin, 2003; Sperling *et al*., 2014), and low food supply limits their abundance on the abyssal ocean floor (Rex *et al*., 2006; Ruhl *et al*., 2008). Availability of oxygen and food influences not only the abundance and diversity of marine animals, but also the structure of ecosystems, for example, by limiting the spatial distribution of active predators (e.g., Levin, 2003; Sperling *et al*., 2014).

Because the biological pump plays such a critical role in structuring modern marine ecosystems, changes over time in the pump strength and remineralization depth have been hypothesized to explain major transitions in the evolution of marine ecosystems. For example, an increase in the remineralization depth due to faster sinking of organic matter has been linked to increased oxygen availability in marine waters during the Cambrian explosion due to either the advent of animal grazing and associated production of fecal pellets (Logan *et al*., 1995) or an increase in the average cell size of marine primary producers (Butterfield, 2009). Increase in the strength of the biological pump (i.e., the flux of organic carbon out of the mixed layer) has been proposed to account for increase in the diversity of marine animals across the Phanerozoic in general as well as the differential diversification of mobile and predatory animals, especially during ecological transitions in the Devonian and Cretaceous periods (Bambach, 1993, 1999; Vermeij, 1995, 2004; Martin, 1996, 2003; Martin & Quigg, 2012; Allmon & Martin, 2014).

Despite the importance of the biological pump for the evolution of marine ecosystems and the numerous hypotheses linking changes in the biological pump to critical transitions in the history of marine animal life, the influence of this critical process on the distribution of nutrients, organic matter, and oxygen in seawater has yet to be explored quantitatively within a three‐dimensional framework accounting for the physical circulation of marine waters. In this study, we explore the influence of the strength of the biological pump and remineralization depth on ocean chemistry in cGENIE, an Earth system model of intermediate complexity. We find that both total nutrient load and remineralization depth have strong effects on the total amount of primary production, the total amount of dissolved oxygen, and the position and extent of oxygen minimum zones. We then attempt to map these findings onto geological time given existing evidence regarding key controls on the biological pump. Finally, we probe the implications of these observations as they relate to current interpretations of secular trends in δ^13^C, patterns in the sedimentary record, and the evolution of animal ecosystems.

## Model Description

We use the cGENIE Earth system model of intermediate complexity (EMIC) (genie.seao2.org) to examine the distribution of oceanic oxygen under a range of nutrient and biological pump conditions. This EMIC efficiently performs simulations on 10^4^‐ to 10^5^‐year timescales relevant to the ocean biogeochemical feedbacks of interest. At its core, cGENIE consists of a 3‐D non‐eddy resolving frictional geostrophic ocean circulation model (Edwards & Marsh, 2005) coupled to the 2‐D energy moisture balance atmospheric model of Weaver *et al*. (2001). The ocean model is based on a 36x36 equal‐area horizontal grid with 16 vertical levels (Cao *et al*., 2009). cGENIE also incorporates a representation of the marine geochemical cycling of carbon and other biologically mediated tracers (Ridgwell *et al*., 2007) including a surface ocean particulate organic matter (POM) export scheme that follows Monteiro *et al*. (2012) with the exception that no nitrogen cycle is included here. In our default configuration, biological remineralization follows the approach of Hotinski *et al*. (2001)in which sulfate and other oxidants are not tracked. When oxygen demand exceeds oxygen supply, an O_2_ deficit is generated, which can be advected and reacted with O_2_ in other grid cells. However, we also explored whether an explicit representation of sulfate reduction coupled with subsequent re‐oxidation of the resulting H_2_S would lead to any qualitative difference in model projections or affect our conclusions (it does not, as we discuss later). Finally, because the model currently lacks an appropriate representation of the burial of organic carbon (and associated nutrient removal), we have employed it in a ‘closed’ configuration in which no mass of any tracer is gained via riverine inputs or lost through sedimentation; ocean–atmosphere gas exchange is allowed.

While the biogeochemical transformations of interest in these simulations are applicable throughout the Phanerozoic, we chose the modern configuration of cGENIE (Cao *et al*., 2009) for simplicity and to remove the additional (small) effects of paleogeography and ocean circulation (Monteiro *et al*., 2012). An annual average wind‐stress field, transformed to a 36 × 36 equal‐area grid is also applied and derived from a pre‐industrial atmospheric level (PAL) *p*CO_2_ experiment (Trenberth *et al*., 1989). As a sensitivity test, we have also configured cGENIE for end‐Permian conditions using the boundary conditions of Kiehl & Shields (2005), because the Permian–Triassic transition is an interval where mass extinction, ocean euxinia, and changes in the biological pump intersect. In both configurations, the ocean is initialized with a modern global average concentration of alkalinity (2363 μeq kg^−1^), dissolved inorganic carbon (DIC) (2159 μeq kg^−1^), and nutrients (2.159 μmol kg^−1^ PO_4_^3−^) (Ridgwell *et al*., 2007; Meyer *et al*., 2008).

We ran a series of numerical experiments to explore the sensitivity of marine oxygen distributions to the marine phosphate inventory and the position of organic carbon remineralization within the water column. In the reference experiments, the ocean is initialized with mean modern phosphate concentration (2.159 μmol kg^−1^ PO_4_^3−^) and all POM is remineralized according to a single e‐folding depth of 589 m. In the modern and end‐Permian sensitivity experiments, the e‐folding depth of remineralization was adjusted to 60, 200, 1000, and 2000 m at each phosphate level specified (0.5, 1, 2, and 5× the modern phosphate reservoir). All simulations were run for 10 kyrs to achieve steady state. Throughout, we maintained the atmospheric CO_2_ concentration and isotopic composition at pre‐industrial values of 278 ppm and −6.5‰, respectively. In the case of the former, this was so as to exclude any changes in climate and hence of ocean circulation that would obfuscate the role of the biological pump in subsequent analysis.

## Results

**Figure 1:**
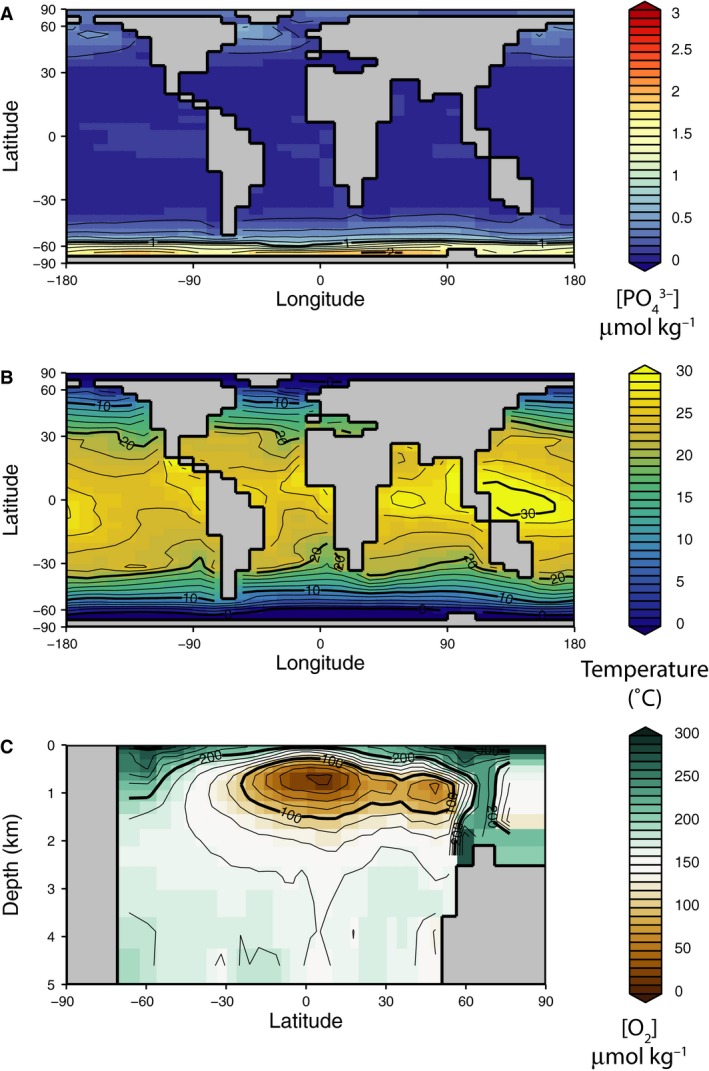
cGENIE model results from the default simulation employing the modern geographic configuration with modern phosphate and e‐folding depth of remineralization. (A) Distribution of dissolved phosphate in the surface ocean. (B) Sea surface temperatures. (C) Dissolved oxygen along an average north–south cross section of the ocean.

Model experiments confirm important roles for both total nutrient availability and the biological pump in controlling the amount of primary production as well as the total amount and spatial distribution of dissolved oxygen in the oceans. Model experiments with modern (1×) marine [PO_4_^3−^] have well‐developed oxygen minimum zones as well as nutrient and oxygen distributions consistent with observations of the modern ocean (Fig. 1). Model experiments varying the phosphate concentration and the e‐folding depth of remineralization demonstrate that both of these variables substantially impact the distribution of food and oxygen in the oceans. Comparison of the modern and end‐Permian results suggests that these first‐order effects are generally independent of paleogeographic configuration.

**Figure 2:**
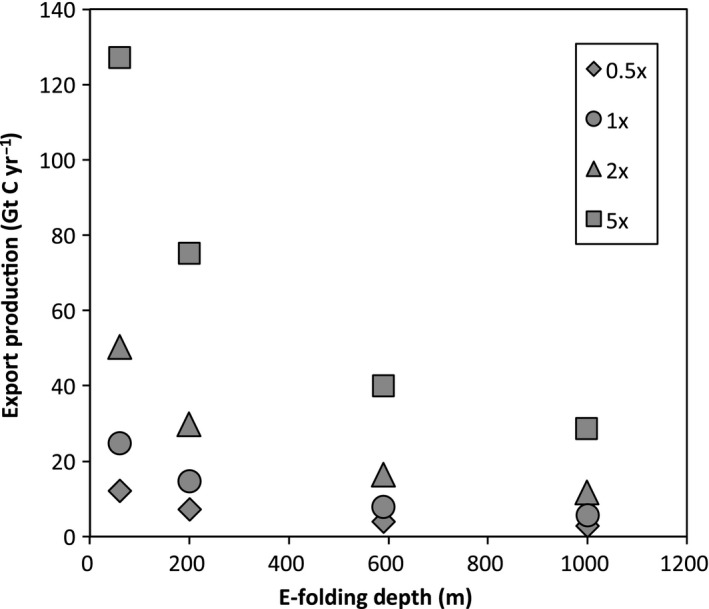
Relationship between e‐folding depth of remineralization, export production, and phosphate concentration. Simulations with shallow remineralization depths and high oceanic phosphate display the greatest export productivities.

Export production is impacted by both total phosphate and the e‐folding depth in model experiments. Model runs with >1× [PO_4_^3−^] show increased export production (Fig. 2), consistent with control by the total availability of phosphate. Export production also varies with the depth of organic matter remineralization. When holding [PO_4_^3−^] constant, simulations with shallower e‐folding depths display higher export production (Fig. 2). Greater nutrient concentrations in simulations with a shallower e‐folding depth result from enhanced remineralization near the surface ocean, allowing for higher primary productivity. Also, with a shallow e‐folding depth, higher [PO_4_^3−^] occurs in upwelling zones and high latitude regions of deep mixing. In scenarios with a deeper remineralization depth than the modern ocean, [PO_4_^3−^] remains very low (<0.5 μmol kg^−1^) in the majority of the surface ocean except for in the polar oceans.

**Figure 3:**
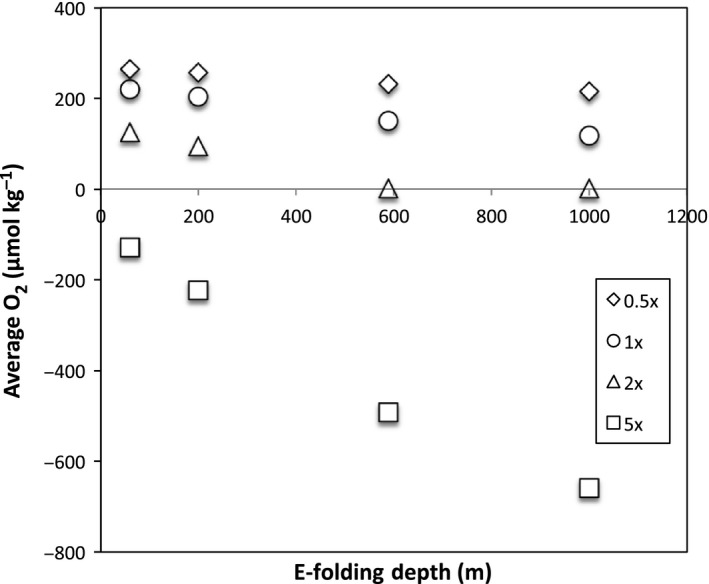
Relationship between e‐folding depth of remineralization, average oxygen concentration, and phosphate concentration. Simulations with high oceanic phosphate display the greatest oxygen depletion.

**Figure 4:**
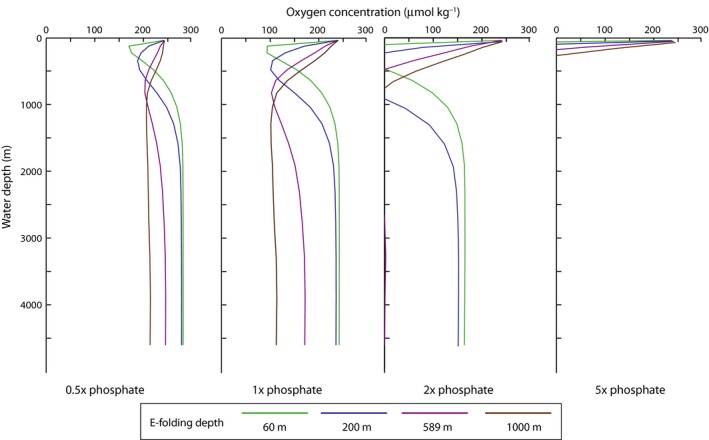
Profiles of oxygen vs. depth for model simulations at 0.5 ×  to 5 ×  modern phosphate (arranged left to right) illustrate that the remineralization depth influences the position of the OMZ and the phosphate inventory influences the degree of oxygen depletion at depth.

Similarly, phosphate content and remineralization depth influence the total oxygen inventory of the ocean. As illustrated in Fig. 3, phosphate exerts greater control than remineralization depth over the total oxygen budget of the ocean. In all cases, increasing the phosphate content of the ocean increases anoxia within the OMZs and the deep ocean. This observation is consistent with nutrient inventory being strongly linked to anoxic events in the Phanerozoic rock record (Meyer *et al*., 2008; Monteiro *et al*., 2012). However, the remineralization depth controls the position of the OMZ (Fig. 4) and thus the amount of continental shelf sediments interacting with anoxic waters.

**Figure 5:**
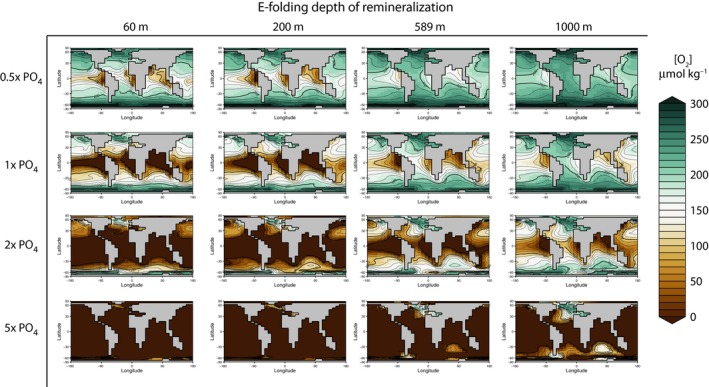
Matrix of model results illustrating the distribution of oxygen in μmol kg^−1^ in the surface ocean (excluding the surface layer, the average concentration over the range ~80–550 m). Maps are arranged according to specified phosphate inventory and remineralization depth.

**Figure 6:**
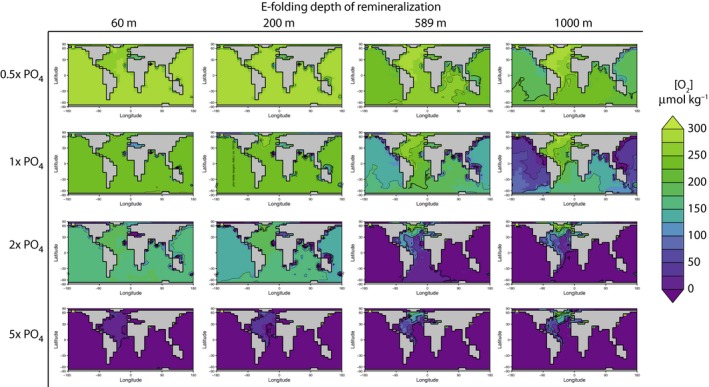
Matrix of model results illustrating the distribution of oxygen in μmol kg^−1^ in the deep ocean. Maps are arranged according to specified phosphate inventory and remineralization depth.

**Figure 7:**
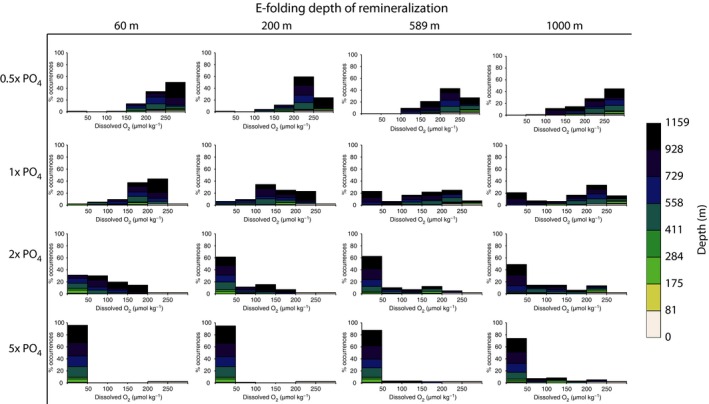
Histograms showing the distribution of oxygen by ocean depth under a range of specified phosphate levels and remineralization depths. The frequency shown on the vertical axis is in grid cells.

Simulations with variable e‐folding depth of mineralization demonstrate the influence of the biological pump on [O_2_] within the ocean interior. In experiments using the modern e‐folding depth (589 m), the oxygen minimum zone is 500–1000 m. Model runs with shallow e‐folding depths (60, 200 m) show reduced depths of the oxygen minimum zones (Fig. 5), whereas greater e‐folding depths cause oxygen depletion at the depth of the modern OMZ and within the deep interior of the ocean (Fig. 6). Comparison of end‐Permian and modern configuration model runs (not shown here) suggests that geography does not have a large effect on these relationships. Interestingly, increasing the remineralization depth causes more widespread dysoxia and anoxia within the deep ocean. This results from the dynamic balance between O_2_ delivery to the deep ocean via downwelling and O_2_ consumption at depth during remineralization. Shallowing the remineralization depth increases the extent of low‐oxygen conditions at continental shelf depths of up to a few hundred meters (Fig. 4). Holding nutrient levels constant while reducing the e‐folding depth of remineralization increases the quantity of nutrients that are liberated near the ocean surface (<200 m water depth), allowing an increase in productivity. This inverse association between e‐folding depth and productivity accounts for the greater number of grid cells of the ocean that contain low oxygen (<50 μmol kg^−1^) as the e‐folding depth is reduced at any given PO_4_^3−^ concentration (Fig. 7).

To examine the impact of the ‘negative O_2_ currency’ scheme on POM remineralization and O_2_ distribution, we contrast the results of model experiments carried out using the negative oxygen (oxygen deficit) approach of Hotinski *et al*. (2001) against results using the same explicit SO_4_^2−^ reduction and H_2_S oxidation scheme as Monteiro *et al*. (2012) and Meyer *et al*. (2008). In the latter, the ocean is initialized with the modern global mean concentration of SO_4_^2−^ (29 mmol kg^−1^), and oxidative remineralization of POM is replaced with sulfate reduction in the absence of oxygen. Dissolved H_2_S generated by this process is advected as a tracer in the ocean circulation model and is oxidized back to sulfate in the presence of oxygen following second‐order reaction kinetics (Zhang & Millero, 1993). We tested both modern and late Permian configurations of the model at 1× [PO_4_^3−^] and 589 m e‐folding depth. The principal difference was that regions experiencing negative [O_2_] in the default configuration displayed ~zero [O_2_] and an [H_2_S] maximum in the sulfate reduction configuration. As a result of the relatively rapid rate of oxidation of H_2_S in the presence of dissolved oxygen (Zhang & Millero, 1993), little difference in the large‐scale distributions of [O_2_] exists between the oxygen deficit and explicit sulfate reduction configurations.

## Discussion and Implications

### Temporal and spatial controls on anoxia

The model results presented here add to existing evidence that the remineralization depth and nutrient inventory have large effects on oxygen availability (Sarmiento *et al*., 1988; Hotinski *et al*., 2001; Meyer *et al*., 2008; Ozaki *et al*., 2011). They confirm previous calculations indicating that total oxygen availability should be inversely related to phosphate inventory (e.g., Fig. 3), which tends to control total primary production and thus oxygen demand over geological timescales (Meyer & Kump, 2008; Meyer *et al*., 2008; Ozaki *et al*., 2011). The simulation experiments further indicate that variation in the remineralization depth can have strong effects on the spatial distribution of low‐oxygen waters within the ocean interior. For example, simply shallowing the e‐folding depth from 589 m (the current value) to 200 m produces as much or more anoxia within the upper 300 m of the water column as doubling the marine phosphate inventory (Fig. 5). In addition, these results add further support to the hypothesis that biogeochemical feedbacks, not changes in physical circulation, have been primary causes of ocean anoxia and euxinia in the geological past (Fischer & Arthur, 1977; Ryan & Cita, 1977). Like the modern ocean, the ocean in this configuration of cGENIE is both chemically stratified and physically well mixed. This chemical stratification is due to the biogeochemical transformations of the biological pump and is sustained due to the continued supply of nutrients to the surface ocean via upwelling (Sigman & Haug, 2003). In these experiments, the overturning circulation remained constant and only phosphate inventory and remineralization depth varied.

Applying these model results to the interpretation of the geological record requires an hypothesis regarding how and why the phosphate inventory and remineralization depth have varied across time. Several lines of evidence suggest that there has been a long‐term trend toward an increase in the remineralization depth. The diversity and abundance of the large, pelagic animals that produce fecal pellets has increased from Proterozoic time toward the present day (Logan *et al*., 1995; Heim *et al*., 2015). The diversity and abundance of marine phytoplankton that produce mineralized shells, which can serve as ballast for marine organic matter, has increased substantially from the Jurassic Period to the present day (Falkowski *et al*., 2004). And the average size of phytoplankton cells has increased from a Precambrian world dominated by prokaryotic phytoplankton to a Phanerozoic world dominated by larger, eukaryotic algae (Falkowski *et al*., 2004).

Although there is less information about how the ocean nutrient inventory has changed through Phanerozoic time, available evidence suggests that ocean phosphate concentrations were relatively constant (Planavsky *et al*., 2010). Because phosphate is more efficiently remineralized under anoxic conditions (Van Cappellen & Ingall, 1996), shallower e‐folding depths during Precambrian and Paleozoic times may have been associated with higher marine phosphate concentrations due to greater prevalence of anoxia at shelf depths where most organic matter is buried. In addition, while the weathering flux of phosphate to the oceans was likely relatively constant, it is possible that the strength of the sedimentary phosphate sink increased slowly over time due to the progressive oxidation of the Earth's surface and the increase in Fe^3+^ sedimentation over time (Hayes & Waldbauer, 2006). This is consistent with the observation of generally waning frequency of anoxia over time, as anoxic events are strongly linked to the marine phosphate inventory via productivity and oxygen demand (Meyer & Kump, 2008; Ozaki *et al*., 2011). In contrast to the poor correspondence between the Phanerozoic record of declining marine anoxia and a hypothesized secular trend of increasing phosphate availability, transient increases in weathering‐derived phosphate coincident with abrupt warming events and the evolution of land plants have been linked to intervals of ocean anoxia (Algeo *et al*., 1998; Meyer & Kump, 2008).

Thus, we hypothesize that the oceans have generally moved from conditions of higher surface ocean phosphate availability and shallower e‐folding depths to lower phosphate availability and deeper e‐folding depths over the Phanerozoic. If the e‐folding depth of organic remineralization has increased over geological time, these trends would have implications for the nature of the sedimentary rock record, the evolution of marine animal ecosystems, and the dynamics of the global carbon cycle. A change in the remineralization depth alone may be sufficient to explain these aspects of the geological record, as large increases in the marine phosphate reservoir ultimately feed back to atmospheric oxygen and prevent long‐term anoxia (Laakso & Schrag, 2014). We discuss these implications below.

### Influence of the evolution of the biological pump on the geological record of shallow‐marine anoxia

The marine sedimentary rock record is characterized by a secular decline in the prevalence of black shales and other laminated, unfossiliferous strata indicative of low‐oxygen conditions in open marine environments (Dunbar & Rodgers, 1957; Pettijohn, 1975; Peters, 2007). Whereas the Proterozoic evidence for ocean anoxia likely reflects low contemporaneous concentrations of oxygen in the atmosphere, the long‐term Phanerozoic decline in the prevalence of black shales is difficult to reconcile with reconstructions of atmospheric *p*O_2_ (Berner & Canfield, 1989; Bergman *et al*., 2004; Berner, 2006, 2009), which do not indicate a monotonic trend toward higher values. Although the redox state of the early Paleozoic oceans may have been impacted by below‐modern levels of atmospheric oxygen (Dahl *et al*., 2010) or reduced thermohaline circulation during intervals of greenhouse climate (Berry & Wilde, 1978), atmospheric oxygen levels were if anything higher than modern during much of the second half of the Paleozoic (Bergman *et al*., 2004; Berner, 2006, 2009). Because the vast majority of the Phanerozoic marine sedimentary rock record exposed on continents reflects deposition in waters less than a few hundred meters deep on continental shelves and within epeiric seaways, we propose that these observations reflect the progressive deepening of the oxygen minimum zone from depths of only 100–300 m to the present values of 500–1000 m.

If correct, this scenario can also account for short‐term episodes of anoxia within longer term periods of better‐oxygenated oceans. Many oceanic anoxic events are associated with the emplacement of large igneous provinces (Arthur *et al*., 1985; Bralower *et al*., 1997; Jenkyns & Wilson, 1999; Wignall, 2001), suggesting a primary control from climate warming due to carbon dioxide release and elevated nutrient delivery to the oceans due to enhanced chemical weathering. Warming, in turn, is typically associated with eustatic sea level rise, and this trangression of anoxic waters has long been hypothesized as a cause of mass extinction (Hallam & Cohen, 1989; Hallam & Wignall, 1997). Warming will also tend to increase the rate of respiration in shallow waters, as metabolic rates typically double for every 10 °C increase in temperature (Peters, 1983), that is, Q_10_ ~ 2. Thus, all else being equal, intense warming of surface waters during the end‐Permian mass extinction (Kiehl & Shields, 2005; Joachimski *et al*., 2012; Sun *et al*., 2012), Paleocene–Eocene Thermal Maximum (Sluijs *et al*., 2006; Zachos *et al*., 2006), and OAE 2 (Bice *et al*., 2006; Forster *et al*., 2007; Jenkyns, 2010), for example, would be expected to decrease the remineralization depth given the magnitude of associated warming (e.g., Zachos *et al*., 2006; Forster *et al*., 2007; Joachimski *et al*., 2012; Sun *et al*., 2012). Indeed, the modeled carbon cycle consequences of assuming a Q_10_ = 2 like function describing POM remineralization in a warm ocean rather than a fixed e‐folding depth have previously been shown to be consistent with pronounced shallow and sharp water column profiles of δ^13^C reconstructed for the early Eocene (John *et al*., 2014). Many of these events are also associated with the extinction of mineralized plankton species and a reduction in their sizes or overall abundances (e.g., Erba & Tremolada, 2004; Tremolada *et al*., 2005), potentially further reducing the remineralization depth. From a modern starting condition, this rise in the position of the OMZ due to the decreased remineralization depth would have a greater effect than the increase in sea level, and the common driver of global warming would help to explain the temporal association between transgression and shallow‐marine anoxia. The decrease in oxygen solubility due to warming and the increase in oxygen demand from warming and weathering‐induced heightened productivity would further amplify this effect. Given the depth of the current oxygen minimum zones, shallowing of the OMZs due to a decrease in the depth of organic remineralization provides a more effective mechanism for explaining the spread of low‐oxygen waters at shelf depths than a simple rise in eustatic sea level.

### Influence of the evolution of the biological pump on secular trends in the structure of animal ecosystems

Biological pump‐driven changes in the prevalence and spatial distribution of marine anoxia can also help to explain an apparent contradiction between Phanerozoic trends in anoxia and hypothesized controls on the structure of animal ecosystems. Numerous paleontologists have noted a long‐term trend in the fossil record toward greater abundance and diversity of large, metabolically active marine animals (e.g., Bambach, 1993, 1999; Vermeij, 1995, 2004; Martin, 1996; Martin & Quigg, 2012). Case studies of gastropods, bivalves, and brachiopods are consistent with this broader hypothesis and suggest more than a ten‐fold increase in animal metabolism through the Phanerozoic (Finnegan *et al*., 2011; Payne *et al*., 2014). Because animals with higher metabolic rates require more food, this trend of increasing metabolic activity has been widely hypothesized to have resulted from an increase in the amount of primary production in the oceans driven by an increase in nutrient availability (e.g., Bambach, 1993, 1999; Vermeij, 1995, 2004; Martin, 1996; Martin & Quigg, 2012). However, our simulation results indicate that any large increase in the marine phosphate reservoir above modern values would likely lead to widespread anoxia. On the other hand, if food supply and marine phosphate levels were far lower than present during Paleozoic time, black shales should be exceedingly rare in those strata due to the resultant low‐oxygen demand in seawater (Fig. 5), opposite to the observed trend in the rock record. A long‐term increase in the remineralization depth of the biological pump provides a mechanism for reconciling these two observations. Under this alternative scenario, the increasing diversity, size, and abundance of active marine animals with high metabolic rates can be explained by an increase in the remineralization depth and a consequent increase in oxygen availability on the continental shelves. Given the sensitivity of oxygen availability in the upper 300 m of the water column to the e‐folding depth, this scenario requires little, if any, change in the total phosphate concentration in seawater and therefore does not require either an increase in total chemical weathering of rocks on land to supply more phosphate over time or even a prolonged dependence on the preferential remineralization of phosphate under anoxic bottom‐water conditions, which should eventually be offset by feedbacks in the oxygen cycle (Van Cappellen & Ingall, 1996; Laakso & Schrag, 2014).

An increase in the remineralization depth can also help to explain the long‐term shift in the dominant phytoplankton from the ‘green’ lineages containing chlorophyll *a* and *b* (e.g., prasinophytes) to the ‘red’ lineages containing chlorophyll *a* and *c* (e.g., coccolithophorids, dinoflagellates, and diatoms). The latter group preferentially uses metal cofactors that are differentially soluble under oxidizing conditions, whereas the former uses cofactors that are differentially soluble under reducing conditions (Quigg *et al*., 2003; Falkowski *et al*., 2004). Increased remineralization depth would have tended to oxygenate ocean bottom waters, thus shifting the relative availability of these metal cofactors. Interestingly, two of the three major red algal lineages also produce mineral ballast—coccolithophorids produce calcite plates (coccoliths), whereas diatoms produce siliceous frustules. These lineages also tend to have larger cell sizes and greater export efficiencies than the green lineages (Katz *et al*., 2004; Butterfield, 2009). To the extent that mineral ballast and cell size influence the strength of the biological pump—and this remains unclear (Wilson *et al*., 2012)—the red lineages may actually help to create and maintain the very biogeochemical conditions that they require through more efficient sinking of their cells through the marine water column.

### Influence of the evolution of the biological pump on stability of the global carbon cycle

Numerical model experiments suggest that variation in the strength of the biological pump may account for many long‐term trends in the chemical and biological structure of the oceans. However, model experiments are necessarily limited in the number of processes explicitly modeled and the range of parameter space that can be explored. Determining the extent to which model experiments actually replicate natural processes requires that they explain a broad range of observations and, ideally, also generate novel, testable predictions. As has long been recognized (e.g., Berry & Wilde, 1978; Logan *et al*., 1995; Butterfield, 2009), variation in the strength of biological pump can account for secular variation in the prevalence of sedimentary rocks exhibiting textural and chemical evidence for deposition under anoxic conditions. Through its control on the spatial distribution of low‐oxygen waters, long‐term increase in the remineralization depth can also account for the timing of major changes in the diversity and ecological structure of marine ecosystems, particularly if the metabolic activity of marine animals has been more limited by oxygen availability than by food supply over the past 500 million years.

**Figure 8:**
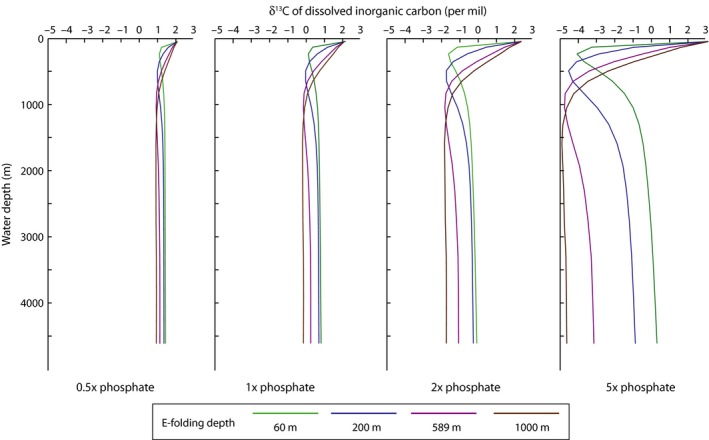
Profiles of δ^13^ C_DIC_ vs. depth for model simulations at 0.5 ×  to 5 ×  modern phosphate (arranged left to right). Similar to the trends in oxygen, the remineralization depth controls the position of the δ^13^C minimum, and the phosphate concentration controls the magnitude of the vertical δ^13^C gradient.

The modeling results also suggest that the depth distribution of carbon isotopes can be used to test this biological pump hypothesis. Although the residence time of carbon in the oceans is much longer than the ocean mixing time (100 ky vs. 1 ky), the concentration and isotope composition of carbon vary laterally and vertically in the oceans due to redistribution of organic matter via the biological pump. Changes in the magnitude of the vertical carbon isotope gradient, and thus the strength and efficiency of the biological pump, have been documented for mass extinctions and subsequent recoveries (Zachos *et al*., 1989; D'Hondt *et al*., 1998; Meyer *et al*., 2011; Song *et al*., 2012, 2013; Luo *et al*., 2014) as well as in relationship to modulating changes in atmospheric *p*CO_2_ over time (Hilting *et al*., 2008; Kwon *et al*., 2009). Our model experiments indicate that the strength of the biological pump and the PO_4_^3−^ content of the ocean set the range of δ^13^C values and the vertical gradient in DIC. At modern nutrient levels, the vertical gradient in δ^13^C in our model is about 2 ‰, approximately the same as the observed gradient in the ocean today. Similar to the spatial distribution of O_2_, the e‐folding depth of remineralization controls the position of the minimum δ^13^C_DIC_ within the upper water column as well as the steepness of the gradient (Fig. 8). With very shallow e‐folding (60, 200 m), the entire gradient is contained within the top 3 layers of the model, or ~280 m. At 1000 m e‐folding depth, the gradient is spread over the top 1000–1500 m. At a doubling of phosphate, the vertical δ^13^C gradient grows to ~4–5‰. At 5× modern PO_4_^3−^, the vertical gradient grows to almost 8‰.

The vertical carbon isotope gradient resulting from the biological pump also is expressed laterally in the surface ocean due to the physical circulation of the ocean. δ^13^C of DIC in gyres is typically more enriched in the heavy isotope and areas of deep mixing or upwelling exhibit more negative δ^13^C _DIC_ values due to the remineralization of organic matter at depth. Both lateral and vertical gradients can be incorporated into the δ^13^C record across environmental gradients via the precipitation of carbonate sediments across space and water depth. Prior to the Triassic, when the majority of preserved sediments were deposited in continental shelf settings, variability in the δ^13^C record could arise partly from capturing these gradients. We propose that one factor that has contributed to the reduction in the amplitude of carbon isotope excursions over the Phanerozoic (Saltzman & Thomas, 2012) is the reduction in the vertical carbon isotope gradient at shelf depths due to an increase in the remineralization depth, due to both the reduced expression of lateral gradients as stratigraphic excursions and the decreased opportunity for the localized burial of highly ^13^C‐enriched or ^13^C‐depleted carbonate sediments.

### Future of the biological pump

As anthropogenic stresses on the ocean mount from climate warming, coastal eutrophication, and overfishing, what changes are expected to ocean biogeochemistry and ecology? The instrumental record suggests an overall decrease in marine oxygen content (Falkowski *et al*., 2011), with expected biological impacts. With ocean deoxygenation, compression of habitat space and greater species interactions are expected (Stramma *et al*., 2010). The deep sea is linked to the surface ocean via the carbon cycle, so changes in delivery of organic carbon would affect the deep‐sea macrofaunal community structure (Ruhl *et al*., 2008). Changing ocean temperature and geochemistry may also influence important zooplankton–microbe relationships in C_org_ degradation in the twilight zone (50–1000 m) (Giering *et al*., 2014). Similar to episodes of transient warming in Earth history, modern climate warming would be expected to impact the biological pump via a reduction in the remineralization depth and increase in nutrient supply. These feedbacks would increase the prevalence of anoxia, especially in shallow waters, and could also alter the air–sea carbon balance (Kwon *et al*., 2009).

## Conclusions

The simple model experiments presented here highlight the ways in which the biological pump has affected marine geochemical and biological patterns through time. If correct, our hypothesis that the remineralization depth of organic matter has generally increased across Phanerozoic time, then these changes may help explain secular trends in anoxia, animal ecosystem structure and diversity, and the prevalence and magnitude of carbon cycle perturbations. Why the different components of the biological pump vary through time and the implications of these changes for long‐term feedbacks between phosphate, anoxia, and oxygen remain to be explained.
